# Supplementary material for: Molecular insights into the regulation of GNPTαβ by LYSET
Source: Nat Commun. 2026 Mar 11;17:3776. doi: 10.1038/s41467-026-70402-6 (PMC13106649; doi:10.1038/s41467-026-70402-6)
Supplement: Supplementary file 1 — Supplementary Information [file 41467_2026_70402_MOESM1_ESM.pdf]

A **B**

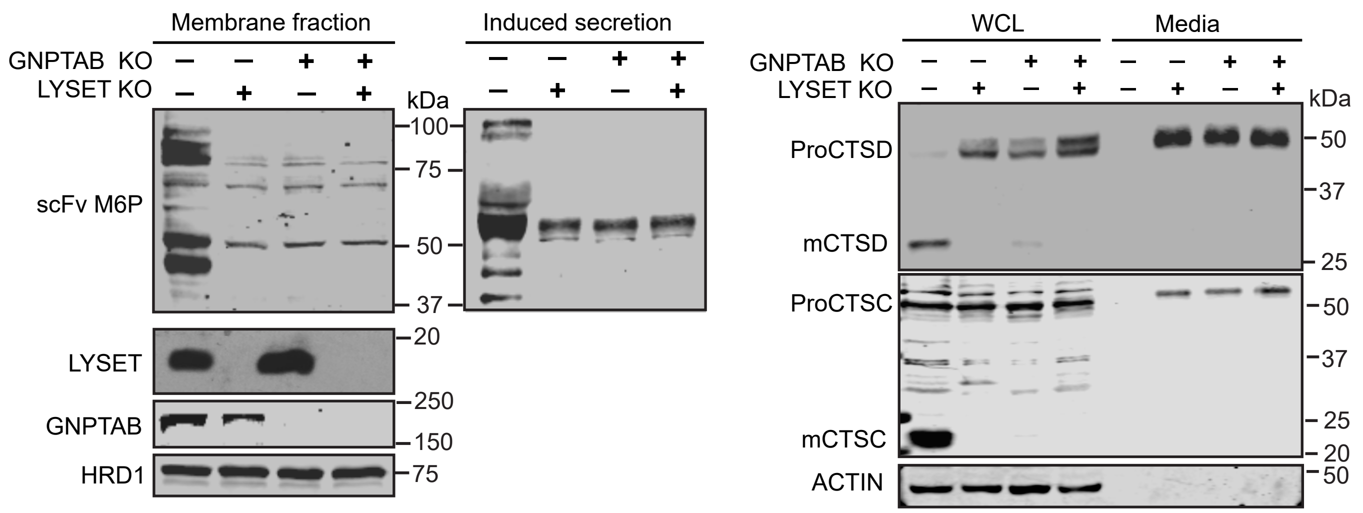

C

Cell line	β-Hex activity (pmol/hr/mg)	α-Gal activity (pmol/hr/mg)	β-Gal activity (pmol/hr/mg)
WT	1746±300	407±41.2	260±51.7
LYSET KO	28±2	4.3±0.6	4.2±0.4
αβKO	29±1.4	4.3±0.3	4.5±0.9
DKO	19±3.1	4.2±0.2	3.9±0.2

D

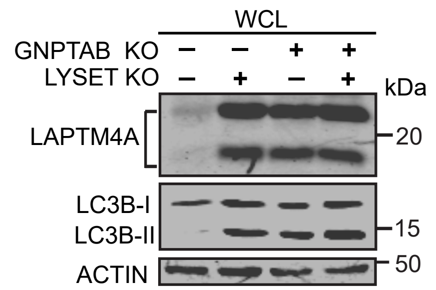

Figure S1: Both GNPTαβ and LYSET are essential for the M6P pathway (related to Fig. 1) (**A**) Detection of M6P modification in HEK293T WT, *LYSET* KO, *GNPTAB* KO, and double KO cells using single-chain antibodies against M6P (scFv M6P). HRD1 was used as a loading control. (**B**) Immunoblot showing CTSC and CTSD maturation in the whole cell lysate (WCL) and conditioned media of HEK293T WT, *LYSET* KO, *GNPTAB* KO, and double KO cells. (**C**) A table showing the enzymatic activity of β-Hex, α-gal, and β-gal in HEK293T WT, *LYSET* KO, *GNPTAB* KO, and double KO cells. Data is presented as mean values +/- standard deviation. N = 3 biological replicates. (**D**) Immunoblot showing the accumulation of LAPTM4A and LC3B-II in HEK293T WT, *LYSET* KO, *GNPTAB* KO, and double KO cells. Source data are provided as a Source Data file.

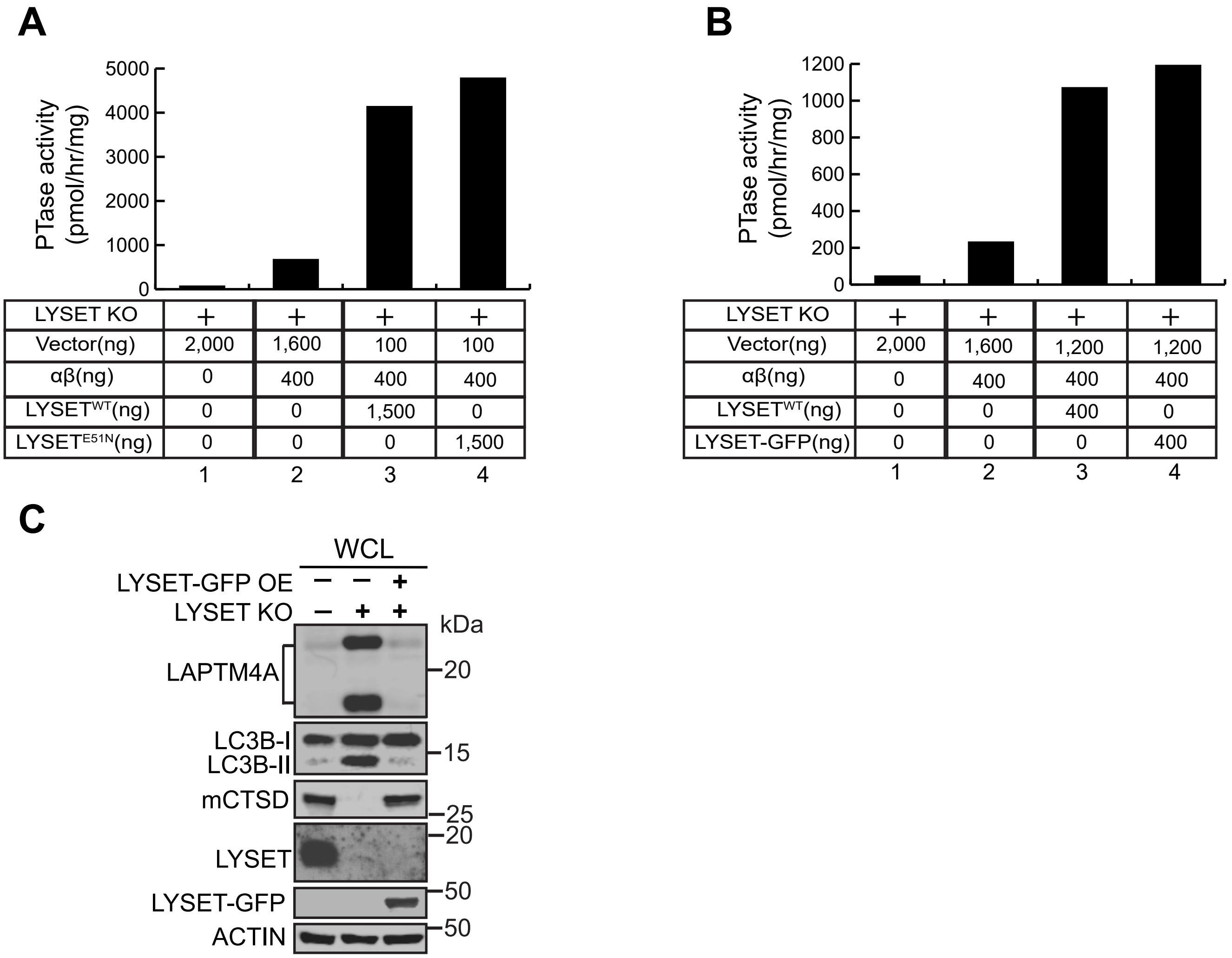

Figure S2: The E51N mutant and LYSET-GFP are functional (related to Fig. 4).

(**A**) The E51N mutant restores GNPTαβ phosphotransferase (PTase) activity to levels similar to WT LYSET. Values represent the average of two assays from two independent transfections. (**B**) LYSET-GFP restores GNPTαβ PTase activity to levels similar to WT LYSET. Values represent the average of two assays from two independent transfections. (**C**) LYSET-GFP rescues the digestion of lysosomal substrates, including LAPTM4A and LC3-II, in *LYSET* KO cells. Source data are provided as a Source Data file.

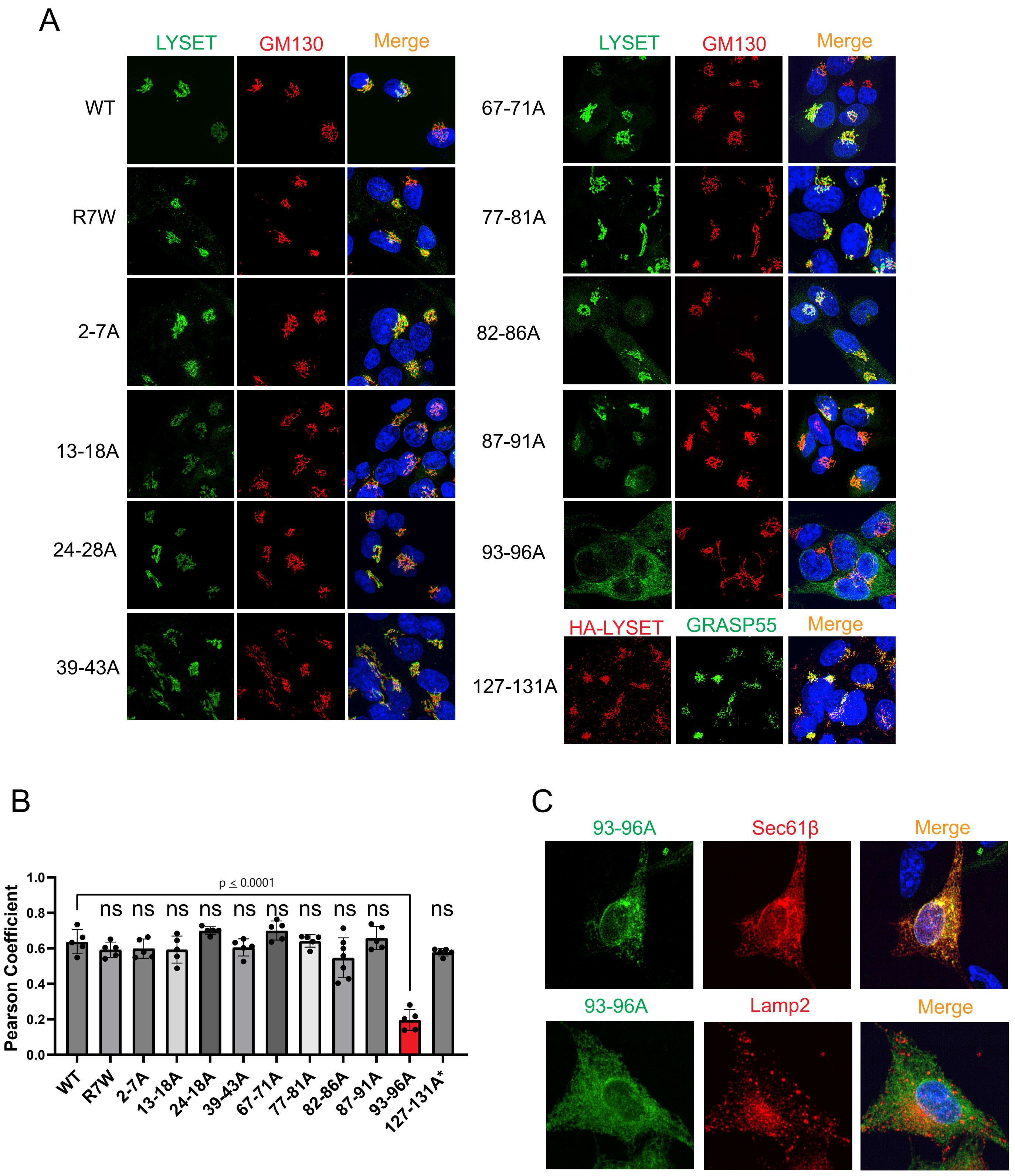

Figure S3: Colocalization analysis of stably overexpressed LYSET mutants with various organelle markers (related to Fig. 5)

(**A**) Immunostaining images showing the localization of the defective LYSET mutants identified in Fig. 5A. (**B**) Pearson's coefficient analysis of (A) between the LYSET mutants and the Golgi marker GM130, or GRASP55. The asterisk denotes the HA-LYSET 127-131A mutant. Each dot represents one cell. Data are presented as mean values +/- standard deviation. Statistical analysis was performed using one-way ANOVA with Dunnett's multiple comparison test. (**C**) The 93-96A LYSET mutant colocalizes with the ER marker Sec61β, but not the lysosome marker LAMP2. Source data are provided as a Source Data file.

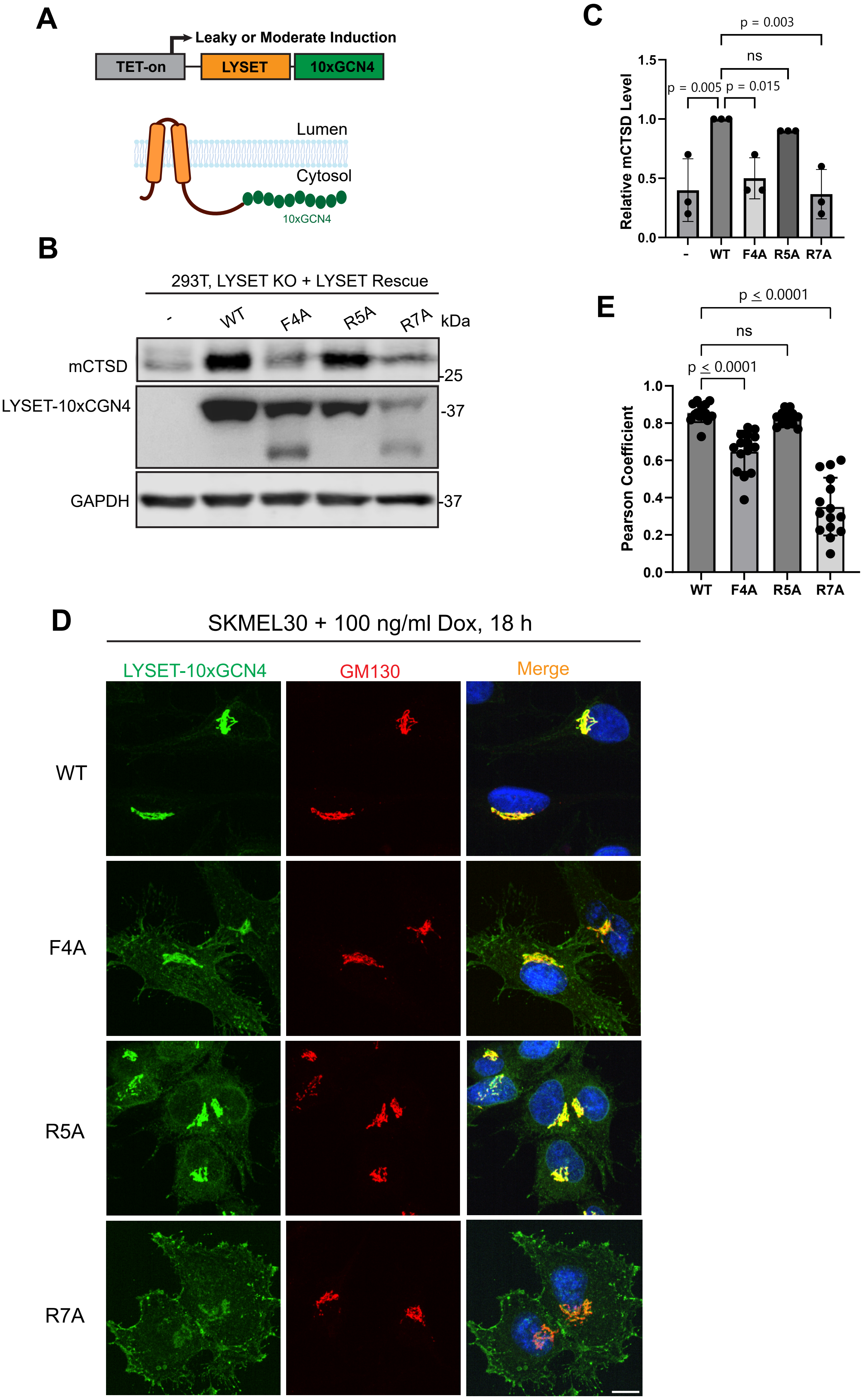

A
Leaky or Moderate Induction
TET-on
LYSET
10xGCN4
Lumen
Cytosol
10xGCN4
B
293T, LYSET KO + LYSET Rescue
-
WT
F4A
R5A
R7A
kDa
mCTSD
LYSET-10xCGN4
GAPDH
-25
-37
-37
C
p = 0.003
ns
p = 0.005
p = 0.015
Relative mCTSD Level
1.5
1.0
0.5
0.0
-
WT
F4A
R5A
R7A
E
p ≤ 0.0001
ns
p ≤ 0.0001
Pearson Coefficient
1.0
0.8
0.6
0.4
0.2
0.0
WT
F4A
R5A
R7A
D
SKMEL30 + 100 ng/ml Dox, 18 h
LYSET-10xGCN4
GM130
Merge
WT
F4A
R5A
R7A

Figure S4: The F^4XXR^7 motif is critical for the function and localization of LYSET (related to Fig. 6)

(**A**) Schematic representation of LYSET tagged with a 10xGCN4 tag. (**B**) Immunoblot analysis of HEK293T *LYSET* KO cells complemented with various LYSET N-terminal mutants under the leaky expression of TET-ON promoter. (**C**) Quantification of (B). Data are presented as mean values +/- standard deviation. Statistical analysis was performed using one-way ANOVA with Dunnett's multiple comparison test, comparing the mutants to WT expressing cells. (**D**) Immunostaining images of LYSET N-terminal mutants in SKMEL30 cells after 18 h of 100 ng/ml doxycycline induction. (**E**) Pearson's coefficient analysis of (D) between LYSET-10xGCN and the Golgi marker GM130. Each dot represents one cell. Data are presented as mean values +/- standard deviation. Statistical analysis was performed using one-way ANOVA with Dunnett's multiple comparison test, comparing the mutants to WT expressing cells. Scale bar: 10 μm. Source data are provided as a Source Data file.

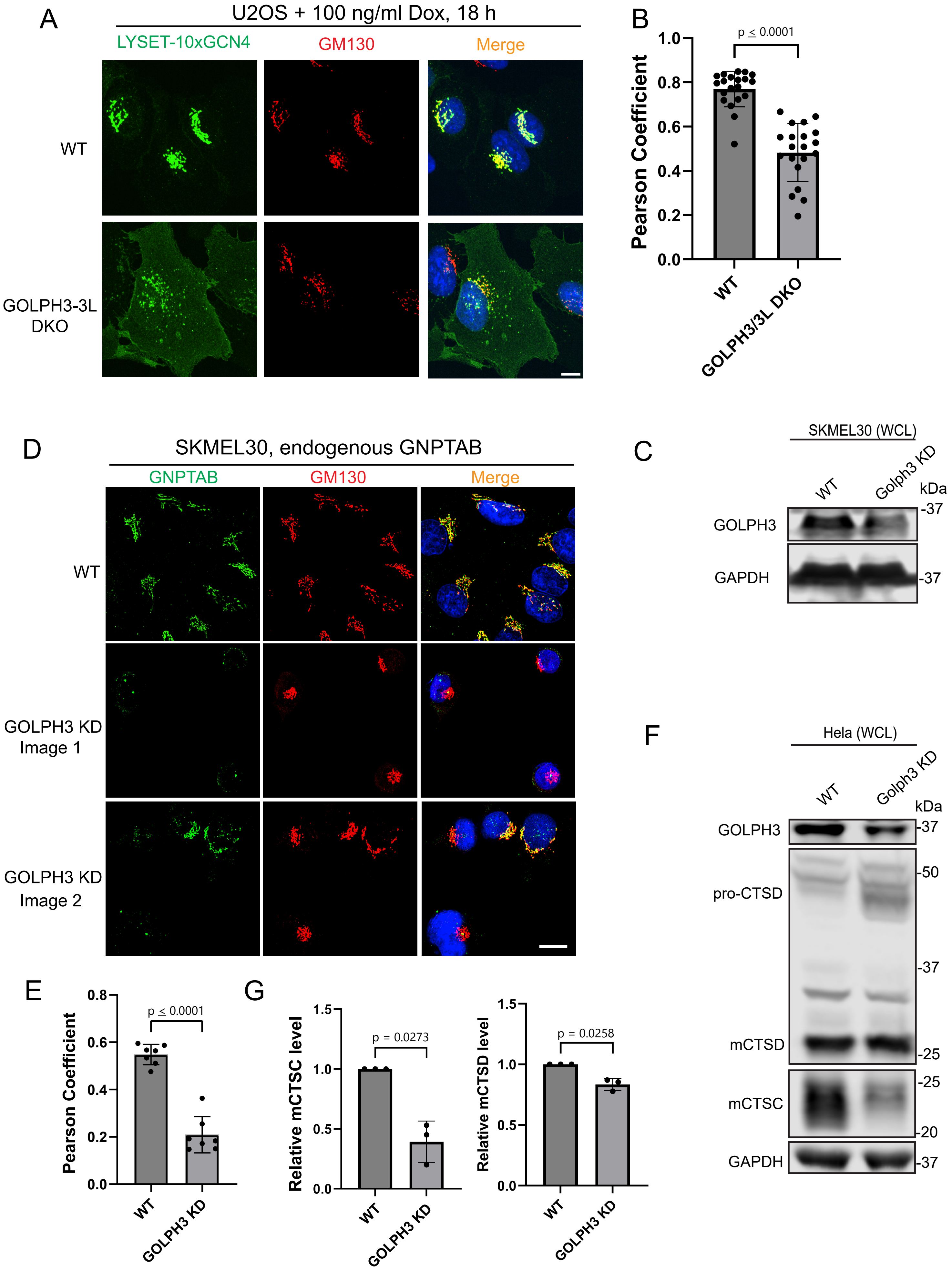
A
U2OS + 100 ng/ml Dox, 18 h
LYSET-10xGCN4
GM130
Merge
WT
GOLPH3-3L DKO
B
p ≤ 0.0001
Pearson Coefficient
1.0
0.8
0.6
0.4
0.2
0.0
WT
GOLPH3/3L DKO
D
SKMEL30, endogenous GNPTAB
GNPTAB
GM130
Merge
WT
GOLPH3 KD Image 1
GOLPH3 KD Image 2
C
SKMEL30 (WCL)
WT
Golph3 KD
kDa
GOLPH3
GAPDH
-37
-37
F
Hela (WCL)
WT
Golph3 KD
kDa
GOLPH3
pro-CTSD
mCTSD
mCTSC
GAPDH
-37
-50
-37
-25
-25
-20
-37
E
Pearson Coefficient
p ≤ 0.0001
0.8
0.6
0.4
0.2
0.0
WT
GOLPH3 KD
G
Relative mCTSC level
p = 0.0273
1.5
1.0
0.5
0.0
WT
GOLPH3 KD
Relative mCTSD level
p = 0.0258
1.5
1.0
0.5
0.0
WT
GOLPH3 KD

Figure S5: GOLPH3 is critical for the M6P pathway in U2OS, HeLa and SKMEL30 cells (related to Fig. 6 & 7)

(**A**) Loss of *GOLPH3/3L* in U2OS cells leads to mislocalization of LYSET-10xGCN4 from the Golgi. LYSET expression was induced with 100 ng/ml doxycycline for 18 hours. **(B**) Pearson's coefficient analysis of (A) between LYSET-10xGCN and the Golgi marker GM130. Each dot represents one cell. Data are presented as mean values +/- standard deviation. Statistical analysis was performed using the two-tailed unpaired T-test with Welch's correction. (**C**) Immunoblot of GOLPH3 knockdown efficiency in SKMEL30 cells. (**D**) Knockdown of *GOLPH3* in SKMEL30 cells results in the loss of endogenous GNPTαβ signal from the Golgi. (**E**) Pearson's coefficient analysis of (C) between GNPTAB and the Golgi marker GM130. Each dot represents one cell. Data are presented as mean values +/- standard deviation. Statistical analysis was performed using the two-tailed unpaired T-test with Welch's correction. (**F**) *GOLPH3* knockdown in HeLa cells causes maturation defects in CTSC and CTSD. (**G**) Quantification of mature CTSC and CTSD (mCTSC and mCTSD) levels from (E). Relative mCTSD levels were calculated as the ratio of mCTSD to total CTSD (mCTSD + proCTSD). Data are presented as mean values +/- standard deviation. N = 3 biological replicates. Statistical analysis was performed using the two-tailed unpaired T-test with Welch's correction. Scale bar: 10 μm. Source data are provided as a Source Data file.

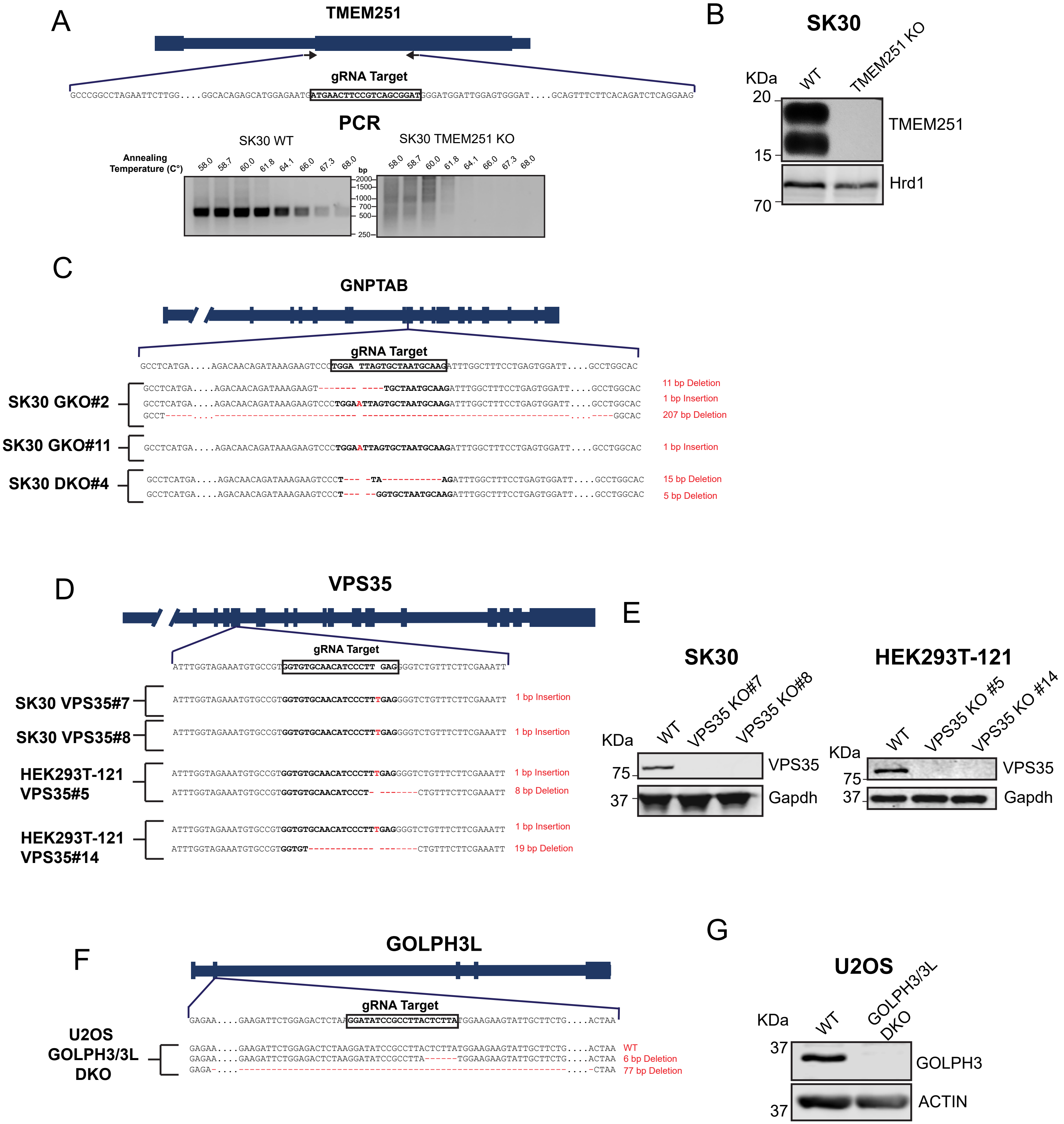

A
TMEM251
gRNA Target
GCCCGGCCTAGAATTCTTGG....GGCACAGAGCATGGAGAATGATGAACTTCCGTCAGCGGATGGGATGGATTGGAGTGGGAT....GCAGTTTCTTCACAGATCTCAGGAAG
PCR
SK30 WT
SK30 TMEM251 KO
Annealing Temperature (C°)
58.0 58.7 60.0 61.8 64.1 66.0 67.3 68.0
bp
2000 1500 1000 700 500 250
B
SK30
KDa
WT
TMEM251 KO
20
15
70
TMEM251
Hrd1
C
GNPTAB
gRNA Target
GCCTCATGA....AGACAACAGATAAAGAAGTCCCTGGA TTAGTGCTAATGCAAGATTTGGCTTTCCTGAGTGGATT....GCCTGGCAC
SK30 GKO#2
11 bp Deletion
1 bp Insertion
207 bp Deletion
SK30 GKO#11
1 bp Insertion
SK30 DKO#4
15 bp Deletion
5 bp Deletion
D
VPS35
gRNA Target
ATTTGGTAGAAATGTGCCGTGGTGTGCAACATCCCTT GAGGGGTCTGTTTCTTCGAAATT
SK30 VPS35#7
1 bp Insertion
SK30 VPS35#8
1 bp Insertion
HEK293T-121 VPS35#5
1 bp Insertion
8 bp Deletion
HEK293T-121 VPS35#14
1 bp Insertion
19 bp Deletion
E
SK30
KDa
WT
VPS35 KO#7
VPS35 KO#8
75
37
VPS35
Gapdh
HEK293T-121
KDa
WT
VPS35 KO #5
VPS35 KO #14
75
37
VPS35
Gapdh
F
GOLPH3L
gRNA Target
GAGAA....GAAGATTCTGGAGACTCTAAGGATATCCGCCTTACTCTTATGGAAGAAGTATTGCTTCTG....ACTAA
U2OS GOLPH3/3L DKO
WT
6 bp Deletion
77 bp Deletion
G
U2OS
KDa
WT
GOLPH3/3L DKO
37
37
GOLPH3
ACTIN

Figure S6: Sanger sequencing and immunoblot analysis confirming the knockout efficiency of the different cell lines created in this study.

(**A**) Schematic representation of the *LYSET* (*TMEM251)* gene and its corresponding gRNA. PCR amplification confirmed the presence of TMEM251 in WT but not in *TMEM251* KO SKMEL30 cells. (**B**) Immunoblot analysis of LYSET protein levels in SKMEL30 WT and *TMEM251* KO cell lines from (A). (**C**) Schematic representation of the *GNPTAB* gene and its gRNA. Sanger sequencing shows the mutations present in SKMEL30 *GNPTAB* KO single colony isolates (#2, #11) and in the *LYSET/GNPTAB* DKO isolate (#4). The DKO clone was generated from the validated LYSET KO SKMEL30 cell line shown in (A–B). (**D**) Schematic of the *VPS35* gene and its gRNA. Sanger sequencing shows the mutations identified in SKMEL30 *VPS35* KO single colony isolates (#7, #8) and HEK293T GNPTAB-3HA *VPS35* KO isolates (#5, #14). (**E**) Immunoblot analysis of VPS35 protein levels in VPS35 KO single colony isolates described in (D). (**F**) Schematic of the GOLPH3L gene and its gRNA. Sanger sequencing results show the mutations in the U2OS *GOLPH3/3L* DKO cell line. Note that GOLPH3L is only partially knocked out. (**G**) Immunoblot analysis of GOLPH3 protein levels in U2OS *GOLPH3/3L DKO* single colony isolates. Source data are provided as a Source Data file.

**Supplemental Table 1: Mammalian cell lines used in this study**		
**Cell lines**	**Description**	**reference/source**
Human HEK293T	CRL-3216	ATCC[42]
Human HeLa	CCL-2	ATCC
Human SKMEL30	SK1980-526	Carey TE et al. (1976) Pubmed ID: 1067619
Human U2OS	HTB-96	From J. Tan, ATCC
Human HEK293T, LYSET KO	LYSET CRISPR-Cas9 knockout	Zhang et al. (2022)
Human HEK293T, GNPTAB KO	GNPTAB CRISPR-Cas9 knockout	Zhang et al. (2022)
Human HEK293T, GNPTAB KO, LYSET KO	LYSET and GNPTAB CRISPR-Cas9 double knockout	This study
Human HEK293T, LYSET KO, GNPTαβ-3V5	LYSET CRISPR-Cas9 knockout, pHAGE2-EF1α-GNPTαβ-3V5-IRES-Blasticidin	This study
Human HEK293T, GNPTAB KO, GNPTαβ-3V5	GNPTαβ CRISPR-Cas9 knockout, pHAGE2-EF1α-GNPTαβ-3V5-IRES-Blasticidin	This study
Human HEK293T, GNPTAB KO, LYSET KO, GNPTαβ-3V5	LYSET and GNPTαβ CRISPR-Cas9 double knockout,	This study

	pHAGE2-EF1α-GNPTαβ-3V5-IRES-Blasticidin	
Human SKMEL30, LYSET KO	LYSET CRISPR-Cas9 knockout	This study
Human SKMEL30, GNPTAB KO	GNPTαβ CRISPR-Cas9 knockout	This study
Human HEK293T, GNPTAB-3HA KI	GNPTαβ-3HA CRISPR-Cas9 knock-in	Zhang et al. (2022)
Human HEK293T, GNPTAB-3HA KI, LYSET KO	GNPTαβ-3HA CRISPR-Cas9 knock-in, LYSET CRISPR-Cas9 knockout	Zhang et al. (2022)
Human HEK293T, LYSET KO, LYSET-GFP	LYSET CRISPR-Cas9 knockout, pHAGE2-EF1α-LYSET-EGFP-IRES-Puro	This study
Human SKMEL30, LYSET KO, LYSET-R7W	LYSET CRISPR-Cas9 knockout, pHAGE2-EF1α-LYSET-R7W-IRES- Blasticidin	This study
Human SKMEL30, LYSET KO, LYSET-2-7A	LYSET CRISPR-Cas9 knockout, pHAGE2-EF1α-LYSET-2-7A-IRES- Blasticidin	This study
Human SKMEL30, LYSET KO, LYSET-13-18A	LYSET CRISPR-Cas9 knockout, pHAGE2-EF1α-LYSET-13-18A-IRES-Blasticidin	This study

Human SKMEL30, LYSET KO, LYSET-24-28A	LYSET CRISPR-Cas9 knockout, pHAGE2-EF1α-LYSET-24-28A-IRES-Blasticidin	This study
Human SKMEL30, LYSET KO, LYSET-39-43A	LYSET CRISPR-Cas9 knockout, pHAGE2-EF1α-LYSET-39-43A-IRES-Blasticidin	This study
Human SKMEL30, LYSET KO, LYSET-67-71A	LYSET CRISPR-Cas9 knockout, pHAGE2-EF1α-LYSET-67-71A-IRES-Blasticidin	This study
Human SKMEL30, LYSET KO, LYSET-77-81A	LYSET CRISPR-Cas9 knockout, pHAGE2-EF1α-LYSET-77-81A-IRES-Blasticidin	This study
Human SKMEL30, LYSET KO, LYSET-82-86A	LYSET CRISPR-Cas9 knockout, pHAGE2-EF1α-LYSET-82-86A-IRES-Blasticidin	This study
Human SKMEL30, LYSET KO, LYSET-87-91A	LYSET CRISPR-Cas9 knockout, pHAGE2-EF1α-LYSET-87-91A-IRES-Blasticidin	This study

Human SKMEL30, LYSET KO, LYSET-93-96A	LYSET CRISPR-Cas9 knockout, pHAGE2-EF1α-LYSET-93-96A-IRES-Blasticidin	This study
Human SKMEL30, LYSET KO, LYSET-127-131A	LYSET CRISPR-Cas9 knockout, pHAGE2-EF1α-3x FLAG- LYSET-127-131A-IRES-Blasticidin	This study
Human HEK293T, LYSET KO, LYSET	LYSET CRISPR-Cas9 knockout, pCW57.1-LYSET-IRES-puro	This study
Human HEK293T, LYSET KO, LYSET-R7W	LYSET CRISPR-Cas9 knockout, pCW57.1-LYSET-R7W-IRES-puro	This study
Human HEK293T, LYSET KO, LYSET-2-7A	LYSET CRISPR-Cas9 knockout, pCW57.1-LYSET-2-7A-IRES-puro	This study
Human HEK293T, LYSET KO, LYSET-8-12A	LYSET CRISPR-Cas9 knockout, pCW57.1-LYSET-8-12A-IRES-puro	This study
Human HEK293T, LYSET KO, LYSET-13-18A	LYSET CRISPR-Cas9 knockout, pCW57.1-LYSET-13-18A-IRES-puro	This study

Human HEK293T, LYSET KO, LYSET-20A	LYSET CRISPR-Cas9 knockout, pCW57.1- LYSET-20A-IRES-puro	This study
Human HEK293T, LYSET KO, LYSET-24-28A	LYSET CRISPR-Cas9 knockout, pCW57.1-LYSET-24-28A-IRES-puro	This study
Human HEK293T, LYSET KO, LYSET-29-33A	LYSET CRISPR-Cas9 knockout, pCW57.1-LYSET-29-33A-IRES-puro	This study
Human HEK293T, LYSET KO, LYSET-34-37A	LYSET CRISPR-Cas9 knockout, pCW57.1-LYSET-34-37A-IRES-puro	This study
Human HEK293T, LYSET KO, LYSET-39-43A	LYSET CRISPR-Cas9 knockout, pCW57.1-LYSET-39-43A-IRES-puro	This study
Human HEK293T, LYSET KO, LYSET-44-48A	LYSET CRISPR-Cas9 knockout, pCW57.1-LYSET-44-48A-IRES-puro	This study
Human HEK293T, LYSET KO, LYSET-49-52A	LYSET CRISPR-Cas9 knockout, pCW57.1-LYSET-49-52A-IRES-puro	This study
Human HEK293T, LYSET KO, LYSET-53-56A	LYSET CRISPR-Cas9 knockout, pCW57.1-LYSET-53-56A-IRES-puro	This study

Human HEK293T, LYSET KO, LYSET-57-60A	LYSET CRISPR-Cas9 knockout, pCW57.1-LYSET-57-60A-IRES-puro	This study
Human HEK293T, LYSET KO, LYSET-62-66A	LYSET CRISPR-Cas9 knockout, pCW57.1-LYSET-62-66A-IRES-puro	This study
Human HEK293T, LYSET KO, LYSET-67-71A	LYSET CRISPR-Cas9 knockout, pCW57.1-LYSET-67-71A-IRES-puro	This study
Human HEK293T, LYSET KO, LYSET-72-76A	LYSET CRISPR-Cas9 knockout, pCW57.1-LYSET-72-76A-IRES-puro	This study
Human HEK293T, LYSET KO, LYSET-77-81A	LYSET CRISPR-Cas9 knockout, pCW57.1-LYSET-77-81A-IRES-puro	This study
Human HEK293T, LYSET KO, LYSET-82-86A	LYSET CRISPR-Cas9 knockout, pCW57.1-LYSET-82-86A-IRES-puro	This study
Human HEK293T, LYSET KO, LYSET-87-91A	LYSET CRISPR-Cas9 knockout, pCW57.1-LYSET-87-91A-IRES-puro	This study
Human HEK293T, LYSET KO, LYSET-93-96A	LYSET CRISPR-Cas9 knockout, pCW57.1-LYSET-93-96A-IRES-puro	This study

Human HEK293T, LYSET KO, LYSET-97-101A	LYSET CRISPR-Cas9 knockout, pCW57.1-LYSET-97-101A-IRES-puro	This study
Human HEK293T, LYSET KO, LYSET-102-106A	LYSET CRISPR-Cas9 knockout, pCW57.1-3xFLAG-LYSET-102-106A-IRES-puro	This study
Human HEK293T, LYSET KO, LYSET-108-111A	LYSET CRISPR-Cas9 knockout, pCW57.1-3xFLAG-LYSET-108-111A-IRES-puro	This study
Human HEK293T, LYSET KO, LYSET-116-118A	LYSET CRISPR-Cas9 knockout, pCW57.1-3xFLAG-LYSET-116-118A-IRES-puro	This study
Human HEK293T, LYSET KO, LYSET-120-121A	LYSET CRISPR-Cas9 knockout, pCW57.1-3xFLAG-LYSET-120-121A-IRES-puro	This study
Human HEK293T, LYSET KO, LYSET-122-126A	LYSET CRISPR-Cas9 knockout, pCW57.1-3xFLAG-LYSET-122-126A-IRES-puro	This study
Human HEK293T, LYSET KO, LYSET-127-131A	LYSET CRISPR-Cas9 knockout, pCW57.1-3xFLAG-LYSET-127-131A-IRES-puro	This study
Human HEK293T, LYSET KO, 3xFLAG-LYSET	LYSET CRISPR-Cas9 knockout, pCW57.1-3xFLAG-LYSET-IRES-puro	This study

Human HEK293T, tet-on GNPTAB-3V5	pCW57.1-GNPTAB-3V5-IRES-puro	This study
Human HEK293T, LYSET KO, tet-on GNPTAB-3V5	LYSET CRISPR-Cas9 knockout, pCW57.1-GNPTAB-3V5-IRES-puro	This study
Human SKMEL30, VPS35 KO	VPS35 CRISPR-Cas9 knockout,	This study
Human HEK293T, GNPTAB-3HA KI, VPS35 KO	GNPTAB-3HA CRISPR-Cas9 knock-in, VPS35 CRISPR-Cas9 knockout	This study
Human HeLa, GOLPH3 KO	GOLPH3 CRISPR-Cas9 knockout	Liu L.,et al. 2018
Human HeLa, GOLPH3 KD	GOLPH3 Knockdown, pLKO	This study
Human HEK293T LYSET KO, tet-on LYSET-10xGCN4	LYSET CRISPR-Cas9 knockout, pCW57.1-LYSET-10xGCN4-IRES-puro	This study
Human HEK293T LYSET KO, tet-on LYSET(F4A)-10xGCN4	LYSET CRISPR-Cas9 knockout, pCW57.1-LYSET(F4A)-10xGCN4-IRES-puro	This study
Human HEK293T LYSET KO, tet-on LYSET(R5A)-10xGCN4	LYSET CRISPR-Cas9 knockout, pCW57.1-LYSET(R5A)-10xGCN4-IRES-puro	This study

Human HEK293T LYSET KO, tet-on LYSET(R7A)-10xGCN4	LYSET CRISPR-Cas9 knockout, pCW57.1-LYSET(R7A)-10xGCN4-IRES-puro	This study
Human SKMEL30, tet-on LYSET-10xGCN4	pCW57.1-LYSET-10xGCN4-IRES-puro	This study
Human SKMEL30, tet-on LYSET(F4A)-10xGCN4	pCW57.1-LYSET(F4A)-10xGCN4-IRES-puro	This study
Human SKMEL30, tet-on LYSET(R5A)-10xGCN4	pCW57.1-LYSET(R5A)-10xGCN4-IRES-puro	This study
Human SKMEL30, tet-on LYSET(R7A)-10xGCN4	pCW57.1-LYSET(R7A)-10xGCN4-IRES-puro	This study
Human SKMEL30, pEF1a IL-2Ra	pEF1a-IL-2Ra-IRES-BSD	This study
Human SKMEL30, pEF1a IL-2Ra-LYSET	pEF1a-IL-2Ra-LYSET-IRES-BSD	This study
Human SKMEL30 VPS35 KO, pEF1a IL-2Ra-LYSET	pEF1a-IL-2Ra-LYSET-IRES-BSD	This study
Human SKMEL30, tet-on LYSET(V97A,Y99A)-10xGCN4	pCW57.1-LYSET(97,99A)-10xGCN4-IRES-puro	This study
Human SKMEL30, tet-on LYSET(V108A,I109A,C110A)-10xGCN4	pCW57.1-LYSET(108-110A)-10xGCN4-IRES-puro	This study

Human SKMEL30, tet-on LYSET(V97A,Y99A V108A,I109A,C110A)-10xGCN4	pCW57.1-LYSET(97,99A,108-110A)-10xGCN4-IRES-puro	This study

**Supplemental Table 2: Mammalian plasmids used in this study**			
**Vector**	**Insert**	**description**	**reference/source**
pSpCas9(BB)-2A-Puro (PX459)		CRISPR-Cas9 knockout	Ran et al. 2013 Addgene, 48139
psPAX2		Lentiviral packaging plasmid	Addgene 12260
pMD2.G		VSV-G envelope	Addgene 12259
Lenti-multi-CRISPR (Mamp194)		CRISPR-Cas9 knockout	Cao et al., 2016 Addgene 85402
AG949 (Mamp372)	scFv M6P	N-terminal IL-2 signal sequence and C-terminal Fc region of Rabbit IgG	University of Geneva, Zhang et al., 2022
Man1A1-GFP	Man1A1	CMV promoter, C-terminal EGFP	
pEGFP-N1	CTNS	CMV promoter, C-terminal GFP	This study
pEGFP-N1	TMEM251	CMV promoter, C-terminal GFP	This study
pcDNA3.1	GNPTAB-V5-6His	CMV promoter	This study
pcDNA3.1	TMEM251	CMV promoter	This study
pcDNA3.1	TMEM251-E41N	CMV promoter	This study

PhageII-pEF1a	TMEM251(88-131)	EF1a promoter, IL2a receptor N-terminal (1-261)	This study
pCW57.1	TMEM251, TMEM251 (F4A), TMEM251(R5A), TMEM251(R7A), TMEM251(97,99A), TMEM251(108-110), TMEM251(97,99,108-110A)	pCW57.1, C-terminal 10xGCN4	This study
