# Supplementary material for: Molecular insights into the regulation of GNPTαβ by LYSET
Source: Nat Commun. 2026 Mar 11;17:3776. doi: 10.1038/s41467-026-70402-6 (PMC13106649; doi:10.1038/s41467-026-70402-6)
Supplement: Supplementary file 2 — Reporting Summary [file 41467_2026_70402_MOESM2_ESM.pdf]

nature portfolio

Corresponding author(s): Ming Li

Last updated by author(s): Jan 12, 2026

Reporting Summary

Nature Portfolio wishes to improve the reproducibility of the work that we publish. This form provides structure for consistency and transparency in reporting. For further information on Nature Portfolio policies, see our Editorial Policies and the Editorial Policy Checklist.

Statistics

For all statistical analyses, confirm that the following items are present in the figure legend, table legend, main text, or Methods section.

n/a	Confirmed	
☐	☒	The exact sample size (*n*) for each experimental group/condition, given as a discrete number and unit of measurement
☐	☒	A statement on whether measurements were taken from distinct samples or whether the same sample was measured repeatedly
☐	☒	The statistical test(s) used AND whether they are one- or two-sided<br>*Only common tests should be described solely by name; describe more complex techniques in the Methods section.*
☒	☐	A description of all covariates tested
☒	☐	A description of any assumptions or corrections, such as tests of normality and adjustment for multiple comparisons
☐	☒	A full description of the statistical parameters including central tendency (e.g. means) or other basic estimates (e.g. regression coefficient) AND variation (e.g. standard deviation) or associated estimates of uncertainty (e.g. confidence intervals)
☐	☒	For null hypothesis testing, the test statistic (e.g. *F*, *t*, *r*) with confidence intervals, effect sizes, degrees of freedom and *P* value noted<br>*Give P values as exact values whenever suitable.*
☒	☐	For Bayesian analysis, information on the choice of priors and Markov chain Monte Carlo settings
☒	☐	For hierarchical and complex designs, identification of the appropriate level for tests and full reporting of outcomes
☒	☐	Estimates of effect sizes (e.g. Cohen's *d*, Pearson's *r*), indicating how they were calculated

Our web collection on statistics for biologists contains articles on many of the points above.

Software and code

Policy information about availability of computer code

Data collection	The Western blotting images were scanned by the Licor Odyssey CLx system. Microscopy was performed with a Dragonfly system (Lecia).
Data analysis	The band intensities of Western were quantified from the raw data files using the Image Studio Ver5.2 software. Images were further cropped or adjusted using ImageJ 1.51 J8(National Institutes of Health). The statistics was done using Microsoft excel (Microsoft, 2016) and Prism (Graphpad, v8.0.2).

For manuscripts utilizing custom algorithms or software that are central to the research but not yet described in published literature, software must be made available to editors and reviewers. We strongly encourage code deposition in a community repository (e.g. GitHub). See the Nature Portfolio guidelines for submitting code & software for further information.

Data

Policy information about availability of data

All manuscripts must include a data availability statement. This statement should provide the following information, where applicable:

- Accession codes, unique identifiers, or web links for publicly available datasets
- A description of any restrictions on data availability
- For clinical datasets or third party data, please ensure that the statement adheres to our policy

N/A

Research involving human participants, their data, or biological material

Policy information about studies with human participants or human data. See also policy information about sex, gender (identity/presentation), and sexual orientation and race, ethnicity and racism.

Reporting on sex and gender	N/A
Reporting on race, ethnicity, or other socially relevant groupings	N/A
Population characteristics	N/A
Recruitment	N/A
Ethics oversight	N/A

Note that full information on the approval of the study protocol must also be provided in the manuscript.

Field-specific reporting

Please select the one below that is the best fit for your research. If you are not sure, read the appropriate sections before making your selection.

[x] Life sciences　[ ] Behavioural & social sciences　[ ] Ecological, evolutionary & environmental sciences

For a reference copy of the document with all sections, see nature.com/documents/nr-reporting-summary-flat.pdf

Life sciences study design

All studies must disclose on these points even when the disclosure is negative.

Sample size	No sample size calculation was used to predetermine the sample size. The number of independent biological replicates for cell based experiments (stated in the Figure legends) were based on experience from similar experiments in our previously published studies and consistent with the current practices in the field. A detailed description of the samples sizes is provided in the manuscript text and figure legend
Data exclusions	No data was excluded from analysis.
Replication	A detailed description of replicates is provided in the text or figure legend. Normally, at least three independent replicates were peformed.
Randomization	For cell based studies, randomization was irrelevant as cells for each experiment were processed and analyzed in parallel.
Blinding	For cell based immunoblotting experiments, operators were not blinded to the experimental groups during collection and analysis as the order of samples was required for data generation.

Reporting for specific materials, systems and methods

We require information from authors about some types of materials, experimental systems and methods used in many studies. Here, indicate whether each material, system or method listed is relevant to your study. If you are not sure if a list item applies to your research, read the appropriate section before selecting a response.

Materials & experimental systems

n/a	Involved in the study	
[ ]	[x]	Antibodies
[ ]	[x]	Eukaryotic cell lines
[x]	[ ]	Palaeontology and archaeology
[x]	[ ]	Animals and other organisms
[x]	[ ]	Clinical data
[x]	[ ]	Dual use research of concern
[x]	[ ]	Plants

Methods

n/a	Involved in the study	
[x]	[ ]	ChIP-seq
[x]	[ ]	Flow cytometry
[x]	[ ]	MRI-based neuroimaging

Antibodies

Antibodies used	The following primary antibodies were used for western blotting in this study: rabbit anti-GFP (1:3000, TP401, Torrey Pines Biolabs), mouse anti-actin (1:5000, Proteintech), mouse anti-GAPDH (1:2000, Proteintech), rabbit anti-CTSD (1:1000, Proteintech), rabbit anti-Golgin160 (1:1000, Proteintech), rabbit anti-LC3 (1:2000, Proteintech), mouse anti-HA (1:500, 16B12, BioLegend), mouse anti-CTSC

April 2023

(1:500, D-6, Santa Cruz Biotechnology), rabbit anti-LAPTM4A (1:1000, HPA068554-1, Millipore-Sigma), mouse anti-LAMP2 (1:1000 H4B4-c, DSHB), rabbit anti-TMEM251 (1:1000, Millipore-Sigma), mouse anti-V5 (1:3000, Invitrogen,460705),rabbit anti-Golgin160 (1:1000, 21193-1-AP, Proteintech), mouse anti-Vps35 (1:500, sc-374372, Santa Cruz Biotechnology), rabbit anti-Vps26 (1:1000, ab23892, Abcam). Rabbit serum containing antibodies against the α-subunit of GNPTab was kindly provided by William Canfield, and the IgG fraction was purified using CaptivA Protein A affinity resin. The generation of single-chain antibodies against M6P was described in Zhang et al. 2021 .The following secondary antibodies were used in this study: goat anti-mouse IRDye 680LT(LI-COR Biosciences,926-68020, 1:10000), goat anti-mouse IRDye 800CW(LI-COR Biosciences,926-32210, 1:10000) , goat anti-rabbit IRDye 680LT(LI-COR Biosciences,926-68021,1:10000), goat anti-rabbit IRDye 800CW(LI-COR Biosciences,926-32211,1:10000). Streptavidin secondary antibodies (IRDye® 800CW Streptavidin, LI-COR Biosciences,926322230, 1:2000).

Validation	Antibodies used in this study are commercially available and validated by the companies. Validation information for each antibody can be found on the manufacturer's website. The GNPTAB antibody was homemade and previously validated.

Eukaryotic cell lines

Policy information about cell lines and Sex and Gender in Research

Cell line source(s)	Cell lines used in this study are listed in Table S1. HEK293 (CRL-1573), HEK293T (CRL-3216), and HeLa (CCL-2) were purchased from ATCC. SK-MEL-30 (SK1980-526) was purchased from Memorial Sloan Kettering Cancer Center
Authentication	Cell lines used in this study were not authenticated by us.
Mycoplasma contamination	All cells were tested negative for mycoplasma.
Commonly misidentified lines (See ICLAC register)	No commonly misidentified cell lines were used in this study.

Plants

Seed stocks	N/A
Novel plant genotypes	N/A
Authentication	N/A
